# Metabolic fatigue in resuscitators using personal protection equipment against biological hazard

**DOI:** 10.17533/udea.iee.v37n2e04

**Published:** 2019-09-19

**Authors:** Francisco Martín-Rodríguez

**Affiliations:** 1 M.Sc, Ph.D. Professor, Centro de Simulación Clínica Avanzada. Facultad de Medicina. Universidad de Valladolid. España Universidad de Valladolid Centro de Simulación Clínica Avanzada Facultad de Medicina Universidad de Valladolid Spain; 2 Unidad Móvil de Emergencias Valladolid I, Gerencia de Emergencias Sanitarias de Castilla y León (SACYL). España. Email: fmartin@saludcastillayleon.es Gerencia de Emergencias Sanitarias de Castilla y León (SACYL) España fmartin@saludcastillayleon.es

**Keywords:** cardiopulmonary resuscitation, personal protective equipment, anaerobic threshold, containment of biohazards, stress, physiological., reanimação cardiopulmonar, equipamento de proteção individual, limiar anaeróbio, contenção de riscos biológicos, estresse fisiológico.

## Abstract

**Objective.:**

To describe the effects of wearing individual protection equipment against biological hazard when performing a simulated resuscitation.

**Methods.:**

Uncontrolled quasi-experimental study involving 47 volunteers chosen by random sampling stratified by sex and professional category. We determined vital signs, anthropometric parameters and baseline lactate levels; subsequently, the volunteers put on level D individual protection equipment against biological hazard and performed a simulated resuscitation for 20 minutes. After undressing and 10 minutes of rest, blood was extracted again to determine lactate levels. Metabolic fatigue was defined as a level of lactic acid above 4 mmol/L at the end of the intervention.

**Results.:**

25.5% of the participants finished the simulation with an unfavorable metabolic tolerance pattern. The variables that predict metabolic fatigue were the level of physical activity and bone mass -in a protective form- and muscle mass. People with a low level of physical activity had ten times the probability of metabolic fatigue compared to those with higher levels of activity (44% versus 4.5%, respectively).

**Conclusion.:**

Professionals who present a medium or high level of physical activity tolerate resuscitation tasks better with a level D individual biological protection suit in a simulated resuscitation.

## Resumo

**Objetivo.:**

Descrever como afeta vestir um equipamento de proteção individual frente a riscos biológicos durante a realização de uma reanimação simulada.

**Métodos.:**

Estudo quase-experimental não controlado no qual participaram 47 voluntários elegidos mediante uma amostragem aleatória estratificado por sexo e categoria profissional. Se realizou uma toma de concreta e de parâmetros antropométricos, assim como uma determinação basal de lactato; posteriormente, os voluntários vestiram um equipamento de proteção individual nível D frente a riscos biológicos e realizaram uma reanimação simulada durante 20 minutos; depois do desvestido e de 10 minutos de repouso se realizou outra extração de sangue para conhecer os níveis de lactato. Se definiu fatiga metabólica se o nível de ácido láctico ao final da intervenção estava por encima de 4 mmol/L.

**Resultados.:**

25.5% dos participantes terminou a simulação com um mal padrão de tolerância metabólica. As variáveis que predizem a fatiga metabólica são o nível de atividade física e a massa óssea -em forma protetora- e a massa muscular. As pessoas com um nível baixo de atividade física tiveram dez vezes a probabilidade de fatiga metabólica comparadas com as de níveis mais altos de atividade (44% versus 4.5%, respectivamente).

**Conclusão.:**

Os profissionais que apresentam um nível de atividade física média ou alta toleram melhor os trabalhos de reanimação com um equipamento de proteção biológica individual nível D, no caso de reanimação simulada.

## Introduction

In "conventional" resuscitation, health personnel must perform cardiopulmonary resuscitation techniques according to protocol. (1) However, the consequences of a cardiorespiratory arrest occurring in highly complex situations, such as an incident with biological hazard, are not known. The risk to health personnel necessarily implies the use of personal protective equipment (PPE). The recent Ebola virus epidemic in West Africa(2) has confronted health systems around the world with an alarming reality of biological hazard situations. It is increasingly common to attend numerous incidents - either provoked or unanticipated - that generate situations of collective emergency in which certain substances with biological hazard are implicated. These are situations that require a highly specialized response; in short: situations that must be handled comprehensively by the Emergency Services. The usual work of emergency teams is *per se* difficult, changing and often unfolding before a complex background. Professionals placed in such scenarios with diverse requirements must have received special attitudes and skills from education and training and in providing materials and resources.

The risk of a situation with biological hazard occurring is percentually low compared with other types of disasters, (3) but, due to its multiple and varied repercussions, the system must be specially prepared and trained. The various PPEs must represent the backbone of protective systems, the prevention of contagion and, by definition, the control of the situation. This type of incidents, despite currently being isolated cases, occur with certain frequency, (4) so we must be prepared to intervene in these scenarios. Most are caused either by accidental situations, or because appropriate safety measures for the handling or transport of certain substances have not been taken.(4) To these accidents we must add the possibility of terrorist acts; a situation that, unfortunately, is happening more frequently, as has been demonstrated in several attempts frustrated by the police worldwide in recent years.(5)

The use of PPE has improved both the assistance to victims and the survival of those involved in chemical or biological incidents, but this type of protection could otherwise reduce a person’s operational capacity. When selecting protective equipment for biological and chemical preparation, a balance must be struck between the degree of protection necessary for the potential hazard in question and the resulting difficulty in carrying out user functions.(6) Performing their work in situations of biological hazard with the necessary level of protection directly affects the physiology of health workers, since it generates significant metabolic fatigue. This metabolic fatigue can increase the risk of accidents with PPE, increase cross-contamination, and lead to hasty termination of the procedures due to physiological stress, among others. Emergency services should contemplate these situations when planning interventions. (7) 

Therefore, the question arises: Are all workers in the emergency services able to tolerate physiologically performing a resuscitation with PPE in the face of biological hazard? To answer this question, we selected a level of lactic acid above 4 mmol/L at the end of the intervention as the parameter for the appearance of metabolic fatigue. (8) The objective of this study was to describe the effects of wearing individual protection equipment against biohazard when performing a simulated resuscitation.

## Methods

*Type of study and sample.* An uncontrolled quasi-experimental study was performed in 2016 including 47 volunteers chosen through random sampling stratified by sex and professional category (doctors and nurses of the Hospital Emergency Services and Prehospital Emergencies) of an opportunity sample of 104 volunteers. We included professionals from the hospital emergency services of the University Clinic and Río Hortega University Hospital in Valladolid and professionals of the prehospital emergencies system of Castilla y León (mobile emergency units of: Palencia, Salamanca and Valladolid, both urban and rural) in Spain. We included voluntary participants between 22 and 65 years. Any volunteer who presented at least one of the following exclusion criteria was rejected: severe motor, visual or hearing impairment, acute phase skin disease, body mass index greater than 40 kg/m^2^, systolic blood pressure below 80 mmHg and baseline heart rate above 150 bpm.

*Environmental conditions and personal protective equipment used.* All participants performed the same simulated clinical case, in the same diaphanous laboratory room of 20 m^2^, with an average controlled temperature of 33.6±4.3ºC and an average controlled humidity of 51.1 ± 1.5%. All PPE elements used in the performance of the practice case conformed with European Community standards and had an instruction manual. Elements are listed in the order of placement: boot covers, protective overall, inner nitrile gloves, hood, FFP3 mask, panoramic and self-ventilated protective goggles and outer nitrile gloves.

**Photo 1:**
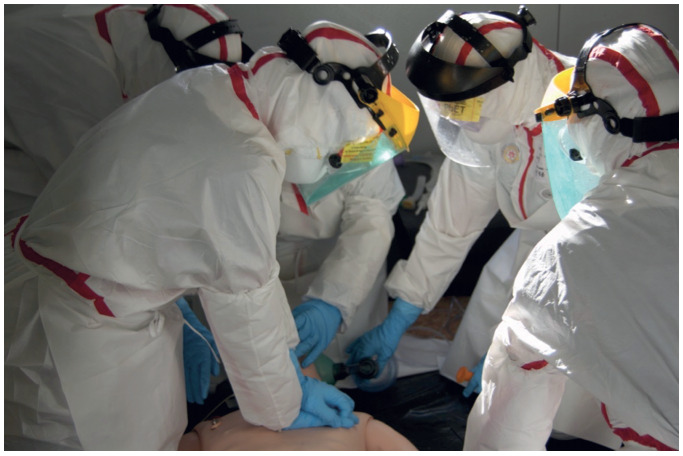
Enactment of the emergency services with biological protection suits (Photo by Francisco Martín-Rodríguez)

*Variables studied and measurement equipment.* An anthropometric study was conducted to assess the following parameters: height, weight, body fat, muscle mass, bone mass, body mass index and total water content. For measuring the volunteers, we used a SECA® model 206 mechanical metric tape and for measuring weight and bio-impedance, we used a Tanita® precision scale model BC-601. The volunteers were asked to sit in a chair, roll up their sleeves and wait 5 minutes calmly, to have their vital signs taken: heart rate, systolic and diastolic blood pressure, respiratory rate, tympanic temperature, total hemoglobin, perfusion index, oxygen saturation and basal glycaemia. For determining systolic blood pressure, diastolic blood pressure and heart rate, we used a SCHILLER brand BP-200 plus meter. The temperature was measured with a tympanic brand BRAUN model ThermoScan PRO 6000 thermometer with ExacTemp technology. The values ​​of total hemoglobin, oxygen saturation and perfusion index were obtained with a MASIMO model Pronto 7 multiparameter monitor, with software version b99e80000004ef796 (2.2.15), and revision version of sensor a83f90f0000c53f2, and glucose levels in blood with an Accu-Chek Mobile meter from Roche®. For determining lactic acid levels, we used an Accutrend® Plus meter from Roche® with a measuring range of 0.8-21.7 mmol/L, with three measurements: baseline determination, just after the volunteer perform the cardiac massage and once the case was concluded after 10 minutes of rest. In addition, each volunteer completed the IPAQ physical activity questionnaire.(9) The test has seven items with high reliability (α = 0.80), suitable for people aged 15 and above. The full version of the questionnaire can be found on the website: www.ipaq.ki.se. The unit of measurement is called METs (unit of measurement of the metabolic index), and corresponds to the sum of the following activities: walking, moderate physical activity and vigorous physical activity. Once the physical activity questionnaire is completed, the volunteers are classified into three levels based on the exercise performed in the last seven days, as follows:(10) *high level*: vigorous physical activity at least 3 days per week achieving a total of at least 1500 METs, or 7 days of any combination of walking, with moderate physical activity and/or vigorous physical activity, achieving a total of at least 3000 METs; *moderate level*: 3 or more days of vigorous physical activity for at least 20 minutes per day, or 5 or more days of moderate physical activity and/or walking at least 30 minutes per day, or 5 or more days of any combination of walking, moderate or vigorous physical activity achieving a total of at least 600 METs; *low or inactive level*: not meeting any of the above criteria.

*Development of the clinical simulation scenario*. All participants in the study had the same information and the same materials and medical devices to solve the same case. The volunteers, guided by a biohazard specialist, had ten minutes to equip themselves completely, following by checking their suits. Once equipped with the PPE, they entered a room with controlled temperature and humidity and had to attend to a convulsing patient with possible biological hazard. After 10 minutes of simulation, the patient suffered a cardiac arrest and the volunteers had to perform a regulated resuscitation during 20 minutes. The total duration of the case inside the laboratory was 30 minutes. Once the practical case was completed, the PPE was taken off under supervision, and 10 minutes after the removal of the PPE, vital signs were taken again.

*Statistical analysis*. The qualitative variables are summarized with their frequency distribution, and the quantitative variables in their mean and standard deviation (SD). In all cases, the distribution of the variable was checked against the theoretical models; and, in the case of asymmetry, we calculated the median and its interquartile range (IQR). The association between qualitative variables was evaluated with the (^2^ test or Fisher's exact test if more than 25% of the expected were less than 5. The behavior of the quantitative variables was analyzed for each of the independent variables categorized by the Student t test. We calculated mean absolute effects and their 95% confidence intervals (95% CI). A logistic regression model was adjusted, in order to evaluate the association of those variables that predicted poor tolerance. This model allowed to identify the relationship between a set of explanatory variables and the probability of control of the variables studied. The calibration capacity of the model was evaluated with the Hosmer and Lemeshow test (p near 1 denoting high calibration). In all hypothesis contrasts, the null hypothesis was rejected with a type I error or alpha error of less than 0.05. The software package used for the analysis was SPSS version 20.0.

*Ethical aspects*. The study was approved on April 6, 2016 by the Clinical Research Ethics Committee of the Río Hortega University Hospital of Valladolid (Spain) with registration code #412016. All volunteers had to read and sign the informed consent document.

## Results

Of 47 participants, 22 were men (46.8%) and 25 women (53.1%), with an average age of 40.2(8.7 years. By profession, 25 were nurses (53.1%) and 22 medical doctors (46.8%); 26 worked in hospital emergency services (55.3%) and 21 in prehospital emergency services (44.6%). On the IPAQ physical activity questionnaire, 25 participants presented a low level of physical activity (53.2%), 14 scored a moderate level of physical activity (29.8%) and 8 presented a level of high physical activity (17%).

Table 1 shows the mean values and standard deviation of the parameters at baseline and according to the final lactic acid values.

**Table 1:** Distribution of vital signs and anthropometric parameters at baseline and according to final lactic acid values

	Baseline	parameters			Final	lactate	
Variables			<4 mmol/L	<4 mmol/L	≥4 mmol/L	≥4 mmol/L	p-value
	Mean	SD*	Mean	SD	Mean	SD	
Age (years)	40.2	8.7	39.3	9.3	42.9	5.8	0.210
Height (cm)	168.9	8.4	168.0	8.4	171.4	8.2	0.225
Weight (kg)	73.5	16.4	70.3	14.0	82.4	19.8	0.026
Body fat (%)	24.0	8.0	22.6	7.2	28.2	9.0	0.034
Muscle mass (%)	52.7	11.5	51.6	10.8	55.9	13.4	0.270
Bone mass (kg)	2.8	0.6	2.7	0.5	2.9	0.7	0.320
Body mass index (kg/m2)	25.5	4.2	24.7	3.6	27.9	5.2	0.024
Total water (%)	55.7	5.5	56.8	4.9	52.5	6.3	0.018
Pulse (bpm)	79.4	12.6	78.8	13.0	81.3	11.4	0.553
Systolic arterial pressure (mmHg)	129.7	13.4	127.4	13.5	136.7	10.5	0.036
Diastolic arterial pressure (mmHg)	83.5	9.4	82.5	9.7	86.3	8.0	0.225
Respiratory rate (rpm)	16.2	1.7	16.1	1.8	16.6	1.5	0.365
Temperature (ºC)	36.5	0.5	36.5	0.6	36.4	0.5	0.945
Saturation (%)	98	1.5	97.9	1.6	98.4	1.2	0.280
Hemoglobin (mg/dl)	13.7	1.4	13.7	1.4	13.7	1.4	0.992
Perfusion (%)	3.6	2.9	3.5	2.7	3.8	3.6	0.755
Glycemia (mg/dl)	114.3	21.8	112.5	18.9	119.8	29.0	0.424
Baseline lactate (mmol/L)	2.3	1.4	2.2	1.2	2.5	2.1	0.587
Lactate during CPR (mmol/L)	9.4	5.2	8.2	5.1	12.7	4.1	0.006
Final lactate (mmol/L)	3.2	1.8	2.3	0.9	5.6	1.7	<0.001
Variation between final and baseline lactate (mmol/L)	1.0	0.40	0.17	1.35	3.03	2.11	<0.001

*Standard Deviation

Table 2 shows that one in four participants concluded the simulation with an unfavourable metabolic tolerance pattern. No statistically significant differences were found in terms of poor metabolic tolerance due to sex or profession variables.

In contrast, statistically significant differences could be observed in the variables of life support training level in environments with biological hazard, where the proportion of subjects with fatigue was greater in the category with basic training (37.5%). By physical activity category performed in the last 7 days, participants with a low level had ten times the probability of metabolic fatigue compared to those with higher levels of activity (44% versus 4.5%, respectively).

**Table 2:** Metabolic fatigue according to study variable categories

Variable	n (%)	p-value
Total (n=47)	12 (25.5)	-
Sex		
Male (n=22)	6 (27.3)	0.797
Female (n=25)	6 (24.0)	
Profession		
Nurse (n=25)	4 (16.0)	0.110
Doctor (n=22)	8 (36.4)	
Workplace		
Hospital emergency dept. (n=26)	8 (30.8)	0.360
Emergency services (n=21)	4 (19.0)	
Training level in life support in biological		
hazard conditions		
Without training (n=6)	1 (16.6)	0.001
Basic training (n=8)	3 (37.5)	
Advanced training (n=33)	8 (24.2)	
Level of physical activity		
Low (n=25)	11 (44.0)	0.008
Moderate (n=14)	1 (7.1)	
High (n=8)	0 (0.0)	
Level of physical activity		
low (n=25)	11 (44.0)	0.002
Moderate to high (n=22)	1 (4.5)	

We adjusted a multivariate logistic regression model in which the variables of professional group, workplace (hospital emergencies or prehospital emergencies), age, physical activity level, body mass index, muscle mass and bone mass were included. The prediction capacity of the model was very good, with an AUC of 0.901 (95% CI 0.81-0.99) and p<0.001.

The variables that predicted metabolic fatigue were the level of physical activity, muscle mass and bone mass. With decreasing physical activity and increasing muscle mass, tolerance worsened, whereas higher bone mass correlated with better tolerance (Table 3).

**Table 3.** Variables for the logistic regression model to predict metabolic fatigue

		95%	CI OR	
Variables	Odds ratio			p-value
		Minimum	Maximum	
Physical activity (intense or moderate compared to low)	0.02	0.00	0.45	0.013
Muscle mass (units)	6.59	1.29	33.75	0.024
Bone mass (units)	0.00	0.00	0.02	0.027

## Discussion

With the generated predictive model, we know *a priori* with excellent reliability which professionals are going to conclude a cardiopulmonary resuscitation with more than 4 mmol/L of lactic acid in blood, an analytical value that characterizes the presence of metabolic fatigue, and value that insinuates the appearance of accidental errors of the workers and decrease in the quality or intensity of the maneuvers necessary for such a critical situation. The results of this study are especially relevant, since they allow establishing the profile of people who would inadequately tolerate the performance of a job with PPE against biological hazard. Knowing the possible behavior of workers at the physiological level, a more efficient selection can be made, and avoiding as much as possible situations of unnecessary risk in the interventions.

The anthropometric parameters behaved in the expected way in the face of physiological stress that requires increased physical activity to generate more bioavailable energy, with increases in muscle mass and water and decrease in body fat.(11) During any moderately intense or highly intense physical exercise (such as a resuscitation with a PPE against biological hazard), the blood pressure is increased to compensate the higher demand for energy. At the end of the exercise, a generalized vasodilatation results, and, as a consequence, a redistribution of blood, lowering blood pressure. After 5-6 minutes of concluding the exercise, the blood pressure decreases to the previous levels at rest, and the blood pressure decreases more to a level below baseline, maintaining this decrease for the following 5-6 hours.(12) The physiological model explains these variations, as a response to intense exercise and the release of catecholamines, leading to peripheral vasoconstriction and to blood redistribution.(13) We found no significant differences by sex, group or study subgroup among subjects with metabolic fatigue.(14) Regarding the variation of lactic acid, during exercise of high intensity and short duration, the organism does not have enough oxygen immediately available, and must get energy through less efficient routes that generate more metabolic waste (glycolytic metabolism).(15) Consequently, high levels of lactic acid form that decrease muscle capacity and the ability to generate energy, causing early fatigue.(16)

In this study, the lactate threshold direct correlated with the physical form of each subject, and revealed substantial differences between people with a high level of physical activity and people with a sedentary lifestyle.(17) In healthy people, we can observe an increase in the levels of lactic acid during exercise of high intensity and short duration (more so in less trained persons). Lactic acid is generated as a metabolic byproduct, becoming recycled as it originates, to a point where the body is unable to recycle lactic acid and it accumulates above 4 mmol/L,(15) exceeding the anaerobic threshold. Consequently, in high levels of lactic acid, the ability to generate energy decreases and muscle capacity decreases, appearing early fatigue.(18) In trained people, this threshold may be higher, and even more important, the capacity to recycle lactic acid is higher, so large quantities cannot accumulate.(19)

Many authors have evaluated the realization of techniques with protection equipment. Szarpak *et al*.(20) studied advanced airway management by paramedical personnel wearing protective suits; the same authors similarly compared the use of intraosseous puncture equipment with suit and without suit,(21) and the performance of conventional vascular access techniques with and without suit.(22) Another study by Szarpak *et al*.(23) evaluated the correct performance of external cardiac massage techniques on a mannequin by professionals in protective suits, evaluating the correct position, depth or quality, among other aspects, but none of these studies evaluated how this physical exertion affected the resuscitators. The study by Stein *et al.,*(24) which analyzes the reaction time of workers carrying PPE and their physiological response, should be highlighted. The authors describe and compare changes in heart rate, venous pH, pCO2, bicarbonate, lactate level, oxygen saturation and temperature. They analyze the variations of these parameters in 19 healthy subjects, in two cases of 20 minutes of exercise without protective equipment, and then the variation during 20 minutes of exercise wearing protective suits. The heart rate and temperature of volunteers in protective equipment were substantially more elevated than in control condition; however, due to the size of the sample, the results were not statistically significant.

If we combine the physiological overload that is caused by the use of specific protection equipment, together with the effort involved in resuscitation tasks, working with biological hazard protection equipment generates discomfort and decreases in the level of attention and response capacity.(24) These circumstances increase the probability of suffering occupational accidents and the risk of exacerbating pre-existing diseases, decreasing effective work time or generating work situations where it is impossible to perform the assigned tasks safely, for the patient, the healthcare worker themselves or the rest of the staff. (25)

Our research is limited to the study of the physiological and anthropometric parameters cited in the methodology, but the usefulness of other parameters such as cortisol, pH or insulin levels, among others, is not discussed. They were discarded from the study due to the complexity involved in measuring them; this limitation has to be taken into account in the study. Broader prospective studies are necessary in order to generalize the results and expand the parameters studied.

Hospital and pre-hospital emergency services must contemplate within their curricular design of competences the handling of incidents with biological hazard, be it as acts of chance or stemming from intentional terrorist acts.(26) Generally, we can affirm that the use of PPE against biological hazard is especially hard and arduous for workers, imposing a burden of additional physiological stress for the intervention.(27) It is easy to demonstrate that work with protective equipment against biological hazard complicates technical procedures; however, so far, no extensive studies have shown that the use of PPE requires highly intense physical effort that prohibits working for large time intervals.(28)

We can conclude that the parameters studied reveal a metabolic pattern of poor physiological tolerance after the use of individual protection equipment level D in the observed sample. Consequently, future studies could derive a predictive rule that allows us to assess which professionals may tolerate and adapt better to work in a biological incident.
